# Exosomes Derived from Adipose Stem Cells Enhance Angiogenesis in Diabetic Wound Via miR-146a-5p/JAZF1 Axis

**DOI:** 10.1007/s12015-024-10685-8

**Published:** 2024-02-23

**Authors:** Dehui Che, Xinjian Xiang, Juan Xie, Zenghong Chen, Qiong Bao, Dongsheng Cao

**Affiliations:** Department of Plastic and Reconstructive, The Second Affiliated Hospital of Anhui Medical University, Hefei, China

**Keywords:** Adipose stem cells, Exosomes, miR-146a-5p, JAZF1, Angiogenesis, Diabetic wound

## Abstract

Chronic trauma in diabetes is a leading cause of disability and mortality. Exosomes show promise in tissue regeneration. This study investigates the role of exosomes derived from adipose stem cells (ADSC-Exos) in angiogenesis. MiRNA-seq analysis revealed significant changes in 47 genes in human umbilical vein endothelial cells (HUVECs) treated with ADSC-Exos, with miR-146a-5p highly expressed. MiR-146a-5p mimics enhanced the pro-angiogenic effects of ADSC-Exos, while inhibitors had the opposite effect. JAZF1 was identified as a direct downstream target of miR-146a-5p through bioinformatics, qRT-PCR, and dual luciferase assay. Overexpress of JAZF1 resulted in decreased proliferation, migration, and angiogenic capacity of HUVECs, and reduced VEGFA expression. This study proposes that ADSC-Exos regulate angiogenesis partly via the miR-146a-5p/JAZF1 axis.

**Graphical Abstract:**

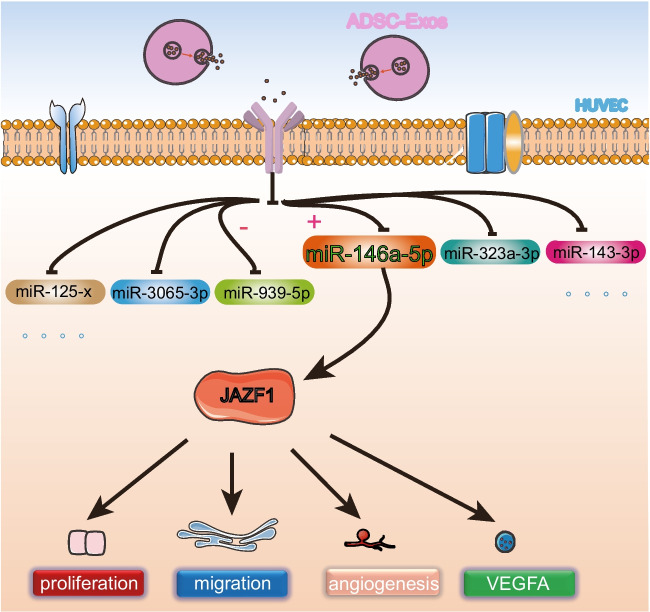

**Supplementary Information:**

The online version contains supplementary material available at 10.1007/s12015-024-10685-8.

## Introduction

Chronic wounds resulting from diabetes pose a considerable challenge in the healing process, inflicting physical pain and emotional distress on the patient, and imposing a significant socioeconomic burden [1]. Vascular dysfunction induced by hyperglycemia is crucial in impeding adequate blood supply to the wounds, contributing to the difficulty in healing diabetic wounds [2, 3]. Growing evidence suggests that promoting neovascularization can enhance nutrient and oxygen delivery to the wound site, consequently expediting wound healing [4, 5].

In recent years, mesenchymal stem cells (MSCs) have demonstrated significant potential in tissue engineering and regeneration. A growing body of evidence supports the association of MSCs with tissue repair and wound healing [6, 7]. Initial studies attributed the therapeutic efficacy of MSCs to their capacity for differentiation into various cell types, such as adipocytes, chondrocytes, or osteoblasts. However, it is now understood that MSCs may primarily exert their effects on paracrine, with secreted factors acting as mediators of tissue repair and wound healing [1]. Exosomes, extracellular vesicles secreted by cells, ranging from 50 to 200 nm in diameter, play a vital role in transporting proteins, RNA, lipids, and other substances to target cells, thus contributing to essential biological function [8–10].

MicroRNAs (miRNAs) are short non-coding RNAs (ncRNAs) comprising approximately 21 ~ 22 nucleotides. They are present in various species and are known to modulate gene expression by targeting the 3’ untranslated region (3’ UTR) of mRNA at the post-transcriptional level [11]. MiRNAs are well-established inducers of translational repression and mRNA decay. The role of exosomes and miRNAs in regenerative medicine is gradually unfolding [12, 13]. However, the precise mechanisms by which exosomes exert their effects through miRNAs in the healing process of traumatic injuries warrant further investigation. In this study, we observed that ADSC-Exos expedited the healing of diabetic wounds in mice. Furthermore, ADSC-Exos stimulated the proliferation, migration, and angiogenesis of HUVECs in a high glucose condition and significantly upregulated the expression of miR-146a-5p. Consequently, we conducted in vitro experiments to validate the functional mechanism of miR-146a-5p in HUVECs behaviour facilitated by ADSC-Exos.

## Results

### Identification and Internalization of ADSC-Exos

**Fig. 1:**
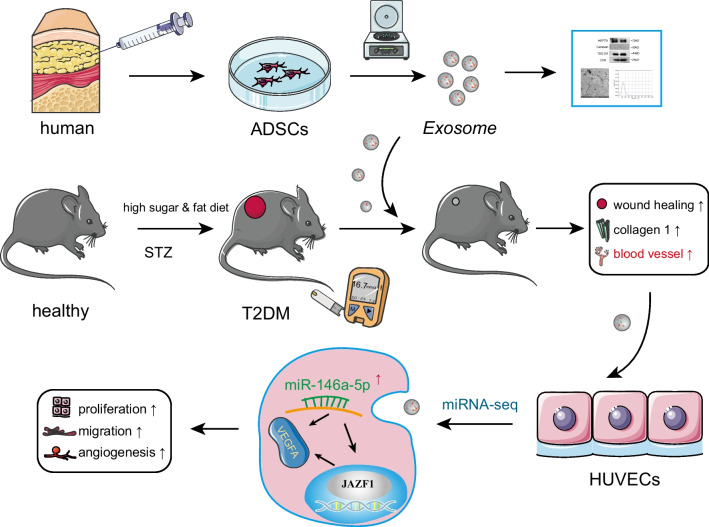
Flow chart of the experiment

**Fig. 2:**
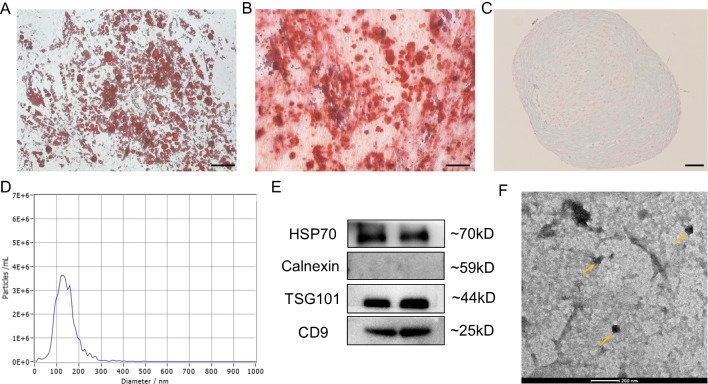
Characterization of ADSC-Exos. **A** Lipogenic differentiation. *N* = 3. Scale bar:100 μm.** B** Osteogenic differentiation. *N* = 3. Scale bar: 100 μm.** C** Chondrogenic differentiation. *N* = 3. Scale bar: 200 μm**. D** NTA for size determination. *N* = 3. **E** Western blot for identification of crucial exosomal proteins. *N* = 3. **F** TEM for visualization of exosomal morphology. *N* = 3. Scale bar: 200 nm

To explore the therapeutic potential of ADSC-Exos on diabetic wounds and elucidate their underlying mechanisms, we isolated stem cells from human adipose tissue and subsequently extracted exosomes (Fig. 1). ADSCs were successfully induced to differentiate into lipid droplets, calcium nodules, and cartilage, displaying characteristics similar to those of MSCs (Fig. 2A, B, C). ADSC-Exos was evaluated using NTA, and the average size was determined to be 127.3 nm (Fig. 2D). Western blot analysis confirmed the presence of surface markers, including TSG101, HSP70, and CD9, without the presence of calnexin (Fig. 2E). Additionally, TEM revealed a cup-shaped morphology of ADSC-Exos (Fig. 2F).

### ADSC-Exos Promote Wound Healing in Diabetic Mice

To ensure biosafety for clinical application, we investigated the biological properties of commercialized Hyaluronic acid Methacrylic anhydride (HAMA). In vitro, ADSC-Exos encapsulated in HAMA demonstrated sustained release (Figure S1A), and HAMA completely degraded within 20 days at 37 °C (Figure S1B). Culturing ADSCs and HUVECs in HAMA, respectively, resulted in a substantial increase in cell number after 2 days, demonstrating excellent biocompatibility and non-cytotoxicity of HAMA (Figure S1C). In vivo, after 1 month of HAMA treatment on mice back wounds, HE staining of the heart, liver, spleen, lung, kidney, and brain tissues showed no histological abnormalities (Figure S1D), confirming the safety of HAMA.

**Fig. 3:**
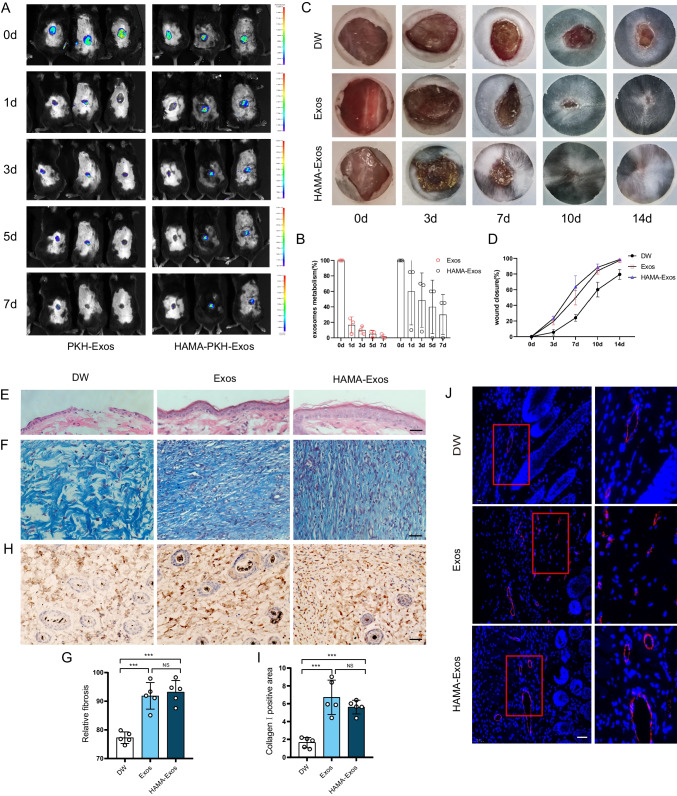
ADSC-Exos promote wound healing in diabetic mice. **A** In vivo imaging technique to assess the metabolism and distribution of ADSC-Exos through subcutaneous injection and trauma-covered HAMA. **B** Fluorescence quantitative analysis of exosomes at various time points. *N* = 3. **C** General observation of diabetic wounds on postoperative days 0, 3, 7, 10, and 14. **D** Statistical analysis of wound closure rate on postoperative days 0, 3, 7, 10, and 14. *N* = 5. **E** HE staining to evaluate the degree of re-epithelialization of wounds. *N* = 5. Scale bar: 100 μm. **F** Masson staining to examine the number and arrangement of collagen fibres in peri-wound tissues. Scale bar: 50 μm. **G** Statistical analysis of Masson. *N* = 5. **H** Immunohistochemistry to detect collagen I synthesis. Scale bar: 50 μm. **I **Quantifcation of the positive areas of the collagen I. *N* = 5. **J** Immunofluorescence detection of the endothelial cell marker VEGFA. *N* = 5. Scale bar: 50 μm. Data are presented as mean ± SD. * p < 0.05, ** p < 0.01, *** p < 0.001

In vivo imaging revealed variations in exosome metabolism among individuals, with no discernible displacement observed. ADSC-Exos subcutaneous injection exhibited significant metabolism by the first day, whereas HAMA-coated ADSC-Exos showed a gradual disappearance (Fig. 3A, B). Both direct subcutaneous injection (twice) and HAMA-Exos surface coverage exhibited enhanced diabetic wound healing (Fig. 3C, D). On day 14, mice were euthanized for skin tissue collection. Exos and HAMA-Exos groups showed more excellent regenerated epidermis and dermis than the DW group (Fig. 3E). Masson staining demonstrated increased collagen formation and denser and more aligned patterns after Exos and HAMA-Exos treatments (Fig. 3F, G). Immunohistochemical analysis showed Exos and HAMA-Exos significantly enhanced collagen I synthesis (Fig. 3H, I). Immunofluorescence staining targeting VEGFA showed increased neovascularization in Exos and HAMA-Exos treated wounds compared to DW (Fig. 3J).

### ADSC-Exos Mitigated Proliferation, Migration and Angiogenesis in HUVECs in vitr*o*

**Fig. 4:**
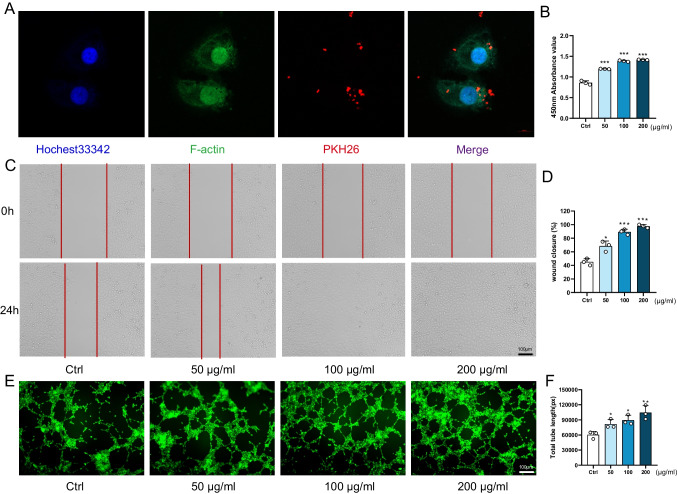
ADSC-Exos mitigated proliferation, migration and angiogenesis in HUVECs. **A** Confocal microscope image captured the uptake of ADSC-Exos by HUVECs after 24 h (Blue: nucleus, Green: cytoskeleton, Red: ADSC-Exos). Scale bar: 10 μm. **B** HUVECs’ proliferation in different treatment groups at 24 h. *N* = 3. **C** Representative images of wound healing of HUVECs in different treatment groups after 24 h. **D** Statistical analysis of wound closure rate. *N* = 3. **E** Representative images of tube formation of HUVECs in different treatment groups. **F** Statistical analysis of total tubule length. *N* = 3. Data are presented as mean ± SD. * p < 0.05, ** p < 0.01, *** p < 0.001

PKH26 labelling was employed to track ADSC-Exos, and the internalization of ADSC-Exos by HUVECs was evident 24 h after treatment (Fig. 4A). Subsequently, HUVECs were exposed to varying concentrations of ADSC-Exos (50, 100, and 200 μg/ml). The results revealed a dose-dependent enhancement in HUVEC proliferation (Fig. 4B), migration (Fig. 4C, D), and angiogenesis (Fig. 4E, F). Based on the results, a final 200 μg/ml concentration was chosen for all subsequent in vitro experiments with ADSC-Exos.

### ADSC-Exos enhance angiogenesis through up-regulating miR-146a-5p

**Fig. 5:**
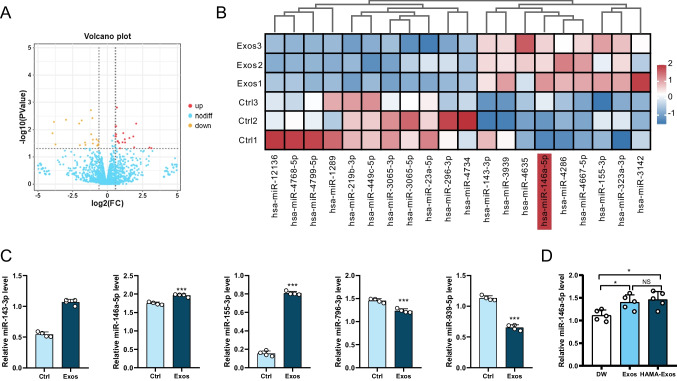
ADSC-Exos enhance angiogenesis through up-regulating miR-146a-5p. **A** Volcano plot depicting changes in miRNA expression before and after HUVECs were treated with ADSC-Exos. Red represents up-regulation, yellow represents down-regulation, and blue represents no significant difference. *N* = 3. **B** Heatmap displaying the top 20 differentially expressed miRNAs. *N* = 3. **C** qRT-PCR validation of the expression of the differential genes miR-146a-5p, miR-143-3p, miR-155-3p, miR-939-5p, and miR-769-3p. *N* = 4. **D** qRT-PCR validation of the expression of miR-146a-5p in the skin tissue of animal models. *N* = 5. Data are presented as mean ± SD. * p < 0.05, ** p < 0.01, *** p < 0.001

To investigate how ADSC-Exos promote angiogenesis, we performed miRNA-seq on ADSC-Exos-treated HUVECs. In total, we detected 1,656 mature miRNAs, and after applying the screening criteria, we identified 47 differentially expressed miRNAs, comprising 23 up-regulated and 24 down-regulated miRNAs (Fig. 5A). Among the top 20 differentially expressed miRNAs, miR-146a-5p showed the highest expression (Fig. 5B). We randomly selected five miRNAs for qRT-PCR to validate the sequencing data, and the results were consistent with the sequencing findings (Fig. 5C). Additionally, we examined the expression of miR-146a-5p in mouse skin tissues and found that the Exos and HAMA-Exos groups exhibited significantly higher expression than the DW group (Fig. 5D). Considering the outcomes, we hypothesized that miR-146a-5p plays a crucial role in the angiogenic effects of ADSC-Exos.

### miR-146a-5p Regulates Proliferation, Migration and Angiogenesis of HUVECs

**Fig. 6:**
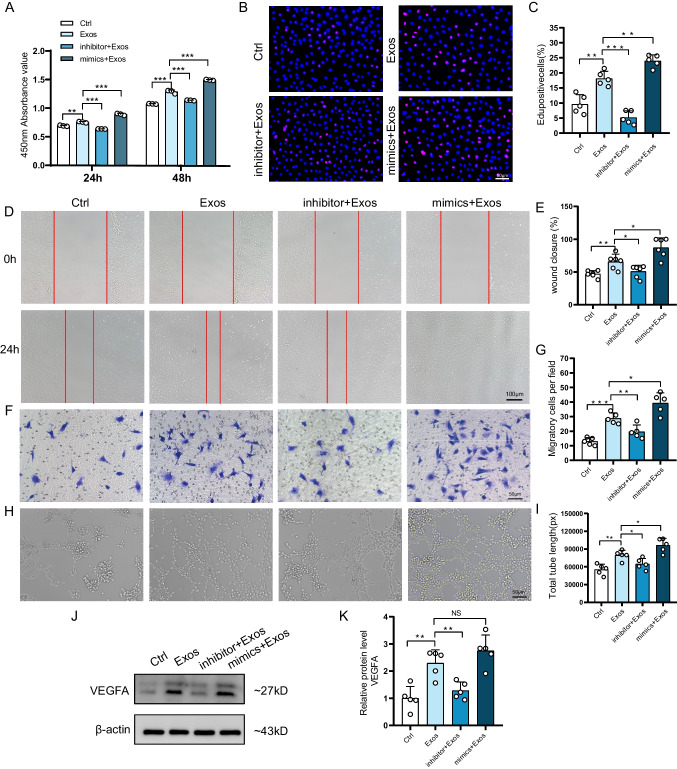
miR-146a-5p regulates proliferation, migration and angiogenesis of HUVECs. **A** CCK-8 assay to assess the proliferative ability of HUVECs in different treatment groups at 24 h, 48 h. *N* = 3. **B** Edu assay fluorescence images showing HUVECs in the proliferative phase in different treatment groups. **C** Percentage of Edu-positive cells in the proliferative phase in different treatment groups. *N* = 5. **D** Wound images of HUVECs in the scratch assay at 0 h and 24 h. **E** Statistical analysis of the wound closure rate of HUVECs in different treatment groups. *N* = 6. **F** Images of migrating HUVECs in the transwell assay in different treatment groups. **G** Statistical analysis of the number of migrating cells in different treatment groups. *N* = 5. **H** Images of tube formation of HUVECs in different treatment groups. **I** Statistical analysis of total tube length of HUVECs in different treatment groups. *N* = 5. **J** Western blotting of VEGFA and β-actin in different treatment groups. **K** Quantitative analysis of the protein level of VEGFA. *N* = 5. Data are presented as mean ± SD. * p < 0.05, ** p < 0.01, *** p < 0.001

To explore the effect of miR-146a-5p on HUVECs, we successfully modulated its expression using miR-146a-5p mimics and inhibitor (Figure S2A). CCK-8 experiments demonstrated that miR-146a-5p inhibitor attenuated the proliferative effect of ADSC-Exos on HUVECs, whereas miR-146a-5p mimics enhanced the impact of ADSC-Exos (Fig. 6A). Edu assay further corroborated cell viability, showing the highest percentage of red fluorescent nuclei in the proliferative phase in the mimics + Exos group and the lowest in the inhibitor + Exos group, compared to the Exos group (Fig. 6B, C). Scratch assay for migration revealed that the trabeculae in the mimics + Exos group were closed mainly at 24 h, while the inhibitor + Exos group had a larger residual trabecular area than the Exos group (Fig. 6D, E). Transwell results were consistent with these findings, with the highest number of HUVECs migrating in the mimics + Exos group (Fig. 6F, G). In the tube formation assay, HUVECs in the mimics + Exos group showed more tubule formation, and the total tubule length in the Exos group was greater than that in the inhibitor + Exos group and the Ctrl group (Fig. 6H, I). Furthermore, ADSC-Exos upregulated the expression of the angiogenesis marker protein VEGFA compared to the Ctrl group, while miR-146a-5p inhibitor impaired this effect (Fig. 6J, K). Overall, these findings indicate that ADSC-Exos regulate the function of HUVECs by affecting the expression of miR-146a-5p.

### JAZF1 Regulates HUVECs Function as a Target Gene of miR-146a-5p

**Fig. 7:**
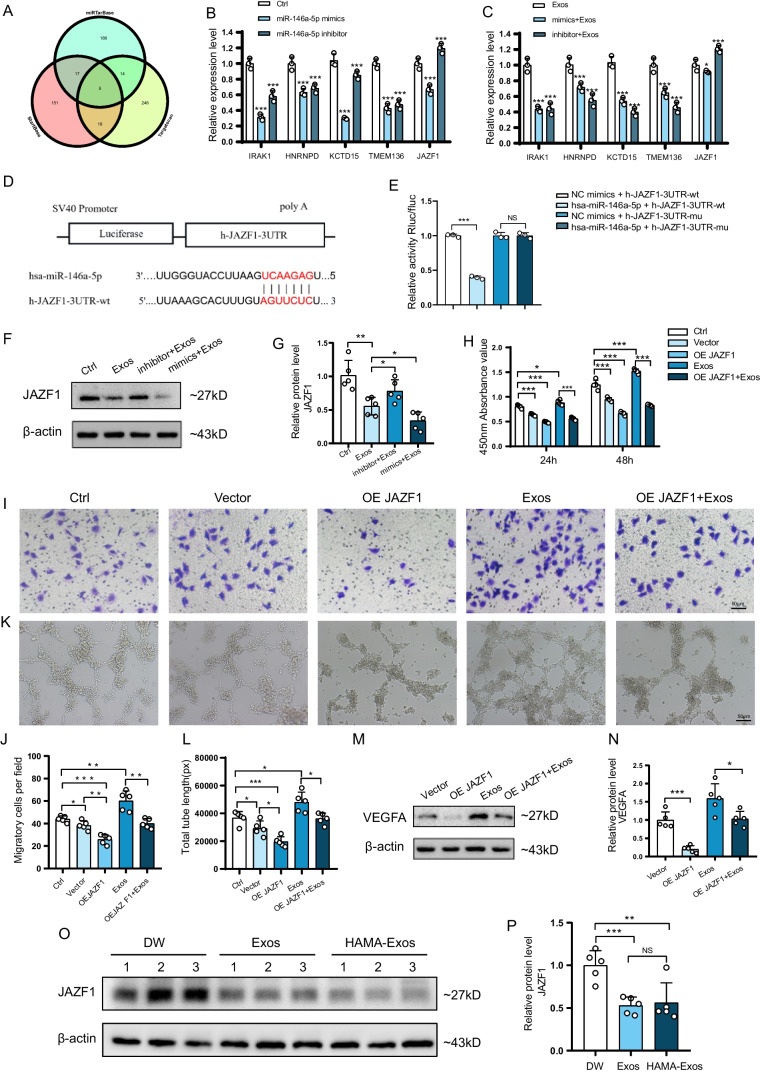
JAZF1 regulates HUVECs function as a target gene of miR-146a-5p. **A** Prediction of miR-146a-5p targets based on TargetScan, StartBase, and miRTarBase. **B** Changes in mRNA levels of predicted target genes after miR-146a-5p overexpression or silencing. *N* = 3. **C** Changes in mRNA levels of predicted target genes after miR-146a-5p overexpression or silencing in the presence of ADSC-Exos treatment. *N* = 3. **D** Bioinformatics prediction of the binding sites of miR-146a-5p and JAZF1. **E** Dual luciferase assay to detect the inhibitory effect of miR-146a-5p on JAZF1. *N* = 3. **F** Western blotting to detect JAZF1 protein expression after miR-146a-5p overexpression or silencing. **G** Quantitative analysis of the protein level of JAZF1. N = 5. **H** Proliferative capacity of HUVECs under different treatment conditions. *N* = 5. **I** Migration images of HUVECs under different treatment conditions. **J** Statistical analysis of the number of migrated cells under different treatment conditions. *N* = 5. **K** Tube formation images of HUVECs under different treatment conditions. **L** Analysis of total tubule length in HUVECs under different treatment conditions. *N* = 5. **M** Western blotting to detect the impact of JAZF1 overexpression on VEGFA protein expression. **N** Quantitative analysis of the protein level of VEGFA. *N* = 5. **O** Western blotting of JAZF1 and β-actin in animal models. **P** Quantitative analysis of the protein level of JAZF1 in animal models. Data are presented as mean ± SD. * p < 0.05, ** p < 0.01, *** p < 0.001

To investigate how miR-146a-5p functions, we used TargetScan, StartBase, and miRTarBase to jointly predict the target genes and identified five possible downstream targets (Fig. 7A): IRAK1, HNRNPD, KCTD15, TMEM136, and JAZF1. qRT-PCR results confirmed significant changes in the mRNA levels of JAZF1 in HUVECs after either miR-146-5p overexpression or silencing (Fig. 7B, C). A binding site was identified between miR-146-5p and JAZF1 (Fig. 7D), suggesting that JAZF1 might be a downstream target of miR-146-5p. The dual luciferase assay further validated this interaction, showing that hsa-miR-146a-5p significantly down-regulated the expression of luciferase of h-JAZF1-3UTR-WT compared with the NC group, confirming the specific binding of miR-146-5p to the predicted target region of JAZF1 mRNA (Fig. 7E). To further characterize the regulatory effects of miR-146-5p on HUVECs in the presence of ADSC-Exos treatment, we treated HUVECs with ADSC-Exos, miR-146-5p mimics + Exos, and miR-146-5p inhibitor + Exos, respectively, and compared the protein-level changes of JAZF1. The results showed that the expression of JAZF1 decreased after miR-146-5p overexpression and increased after miR-146-5p silencing (Fig. 7F,  G).

To study the role of JAZF1 in the behaviour of HUVECs, we transfected HUVECs with the JAZF1 pcDNA3.1. The mRNA and protein expression levels increased significantly (Figure S2B, C). The results of CCK-8, Transwell and Tube formation assays showed that after transfection with OE JAZF1, the proliferative ability, migratory power, and angiogenic potential of HUVECs were reduced. Additionally, OE JAZF1 weakened the effect of ADSC-Exos on HUVECs (Fig. 7H, I, J, K, L). Western blotting showed that after overexpression of JAZF1, the protein level of VEGFA was significantly decreased (Fig. 7M, N). Furthermore, protein assays were conducted on traumatic skin tissue samples obtained from three distinct groups of animal models. The results revealed a noteworthy reduction in the expression of JAZF1 in both the Exos- and HAMA-Exos-treated groups compared to the DW group (Fig. 7O, P). This observation serves to revalidate our earlier discoveries.

## Discussion

Previous studies have confirmed the therapeutic efficacy of MSC-derived exosomes in addressing challenging wound-healing cases [14, 15]. In a clinical study published in 2023[16], notable advancements were observed in the treatment of chronic venous ulcers that were unresponsive to conventional therapies. The study demonstrated enhanced therapeutic outcomes through the utilization of exosomes derived from the patient’s autologous serum. These improvements were evidenced by increased microvascular proliferation in regenerating tissue and augmented formation of granulation tissue. Extracellular vesicles derived from ADSCs confer a protective effect against muscle injury [17]. This protective mechanism operates through interactions with tissue microvessels and muscle cells, demonstrating favorable therapeutic effects in a mouse hindlimb ischemia model. These findings affirm the promising potential of cell-free therapy within the realm of tissue regeneration. However, the underlying mechanism of action still needs to be understood. This study aimed to assess the primary role of ADSC-Exos in promoting vascular regeneration in difficult-to-heal wounds associated with diabetes. Our findings demonstrate that both subcutaneous injection of ADSC-Exos and the application of HAMA piggybacked ADSC-Exos resulted in superior therapeutic effects on skin injury healing in a mouse model of diabetes (Fig. 3C, D). Additionally, these treatments significantly enhanced neovascularization (Fig. 3J). Furthermore, in vitro experiments proved that ADSC-Exos promoted the proliferation, migration, and vascular regeneration of HUVECs in a high glucose (Fig. 4). Importantly, we identified the involvement of the miR-146a-5p/JAZF1 axis as a partial mediator of this mechanism.

The functionality of exosomes is heavily influenced by their origin, isolation method, administration route, dosage, and target tissue. Following systemic injection, exosomes mainly accumulate in the spleen and liver, with clearance from the animals observed within 6 h after administration [18, 19]. Local administration to injured tissues improves exosome targeting; nonetheless, those exosomes unaffected by proteases or pH changes exhibit a shorter lifespan due to rapid uptake by tissue cells, including immune and endothelial cells [20, 21].

One of the conventional approaches for drug treatment in trauma involves subcutaneous injection, providing rapid drug action through the skin but lacking sustained drug delivery. Another method, spraying, covers a larger area but results in drug loss. Hydrogels have emerged as a suitable option for tissue engineering and regenerative medicine due to their adjustable physicochemical properties, mimicking the natural extracellular matrix. In recent years, hydrogel drug delivery has gained significant attention for creating a moist environment conducive to wound repair, ensuring long-term drug delivery and better wound coverage [22]. In this study, we explored ADSC-Exos delivery using subcutaneous injection and HAMA to cover the wound, both exhibiting enhanced therapeutic effects. However, considering the drawbacks of repeated subcutaneous administration, such as increased patient discomfort and medical staff workload, as well as potential disruptions to wound healing, we propose using hydrogel for trauma coverage. HAMA can be solidified within 5 s of UV light irradiation, effectively isolating the injury from the external environment, reducing infection risks, and minimizing patient discomfort. As such, the hydrogel is a promising exosome delivery carrier. Hence, before clinical application, it is crucial to conduct additional biosafety evaluations of tissue engineering constructs based on HAMA to address potential biosafety concerns. Following 1 month of continuous HAMA treatment on the backs of mice, HE staining of the heart, liver, spleen, lungs, kidneys, and brain tissues did not reveal any apparent abnormalities (Figure S1D), providing essential safety insights.

Our research group previously investigated miR-139-5p and revealed its influence on hemangioma stem cells’ (HemSC) proliferation, migration, and adipogenesis by modulating the IGF-1/IGF-1R pathway [23]. miRNAs derived from stem cell exosomes also play a pivotal role in modulating the transferability of stem cell properties to recipient cells [24]. In this study, we observed significant changes in the expression of 47 miRNAs in HUVECs after treatment with ADSC-Exos (Fig. 5A), of which the expression of miR-146a-5p was significantly higher than the other genes (Fig. 5B). Meanwhile, we performed miR-146a-5p on the skin tissues of animal models and found that the expression was significantly higher in the Exos and HAMA-Exos groups (Fig. 5D). Consequently, we hypothesized that miR-146a-5p might be the primary gene affecting wound healing.

ADSC-Exos have been reported to exhibit a substantial enrichment of miR-146a-5p [25, 26]. Serena et al. conducted an analysis of the small RNA expression profile in exosomes secreted by human bone marrow- and adipose-derived mesenchymal stem cells [25]. The study revealed minimal intra- and inter-sample variability in MSCs, suggesting low intrinsic differences. However, notable distinctions emerged when comparing exosomes, indicating that the tissue-specific microenvironment may influence the sorting of MSC-derived exosomes. Intra-sample variability implies that inherent factors within the cells, such as their differentiation state, may dictate the signals conveyed by the cells. An extensive literature review revealed that reduced expression of miR-146a-5p triggers inflammation, which is closely associated with the development of diabetic vascular complications [27, 28]. Barutta et al. conducted a case–control study involving 447 patients with type 1 diabetes mellitus. They found a significant negative correlation between miR-146a-5p levels and chronic diabetic complications, particularly cardiovascular disease and retinopathy [29].

To explore the functional effects of miR-146a-5p on HUVECs, we employed miR-146a-5p mimics and inhibitors. Our results demonstrated that miR-146a-5p mimics enhanced the promotional impact of ADSC-Exos on HUVEC proliferation, migration, and angiogenesis. Conversely, miR-146a-5p inhibitors exhibited the opposite effect (Fig. 6). Similar findings were reported in breast cancer studies, where miR-146a-5p demonstrated pro-angiogenic effects [30]. Su et al. showed that miR-146a/b promoted endothelial progenitor cells’ proliferation, migration, and angiogenic capacity by down-regulating TRAF6 and IRAK1 expression [31]. Another study demonstrated that BMSC-derived exosomes significantly enhanced angiogenesis in HUVECs in vitro, attributed to increased pro-angiogenic miR-146a expression and inhibition of Smad4 and NF2 proteins [32]. Our experimental results align with the abovementioned studies, confirming that miR-146a-5p plays a critical regulatory role in vascular regeneration.

In this investigative endeavor, directed at unraveling the influence of miR-146a-5p on HUVECs functionality, a meticulous exploration ensued to identify potential downstream targets. Employing TargetScan, StartBase, and miRTarBase databases, we uncovered five identical predicted targets, namely IRAK1, HNRNPD, KCTD15, TMEM136, and JAZF1 (Fig. 7A). Subsequently, we employed qRT-PCR and a dual luciferase assay to meticulously validate these predictions, conclusively affirming JAZF1 as a direct target of miR-146a-5p (Fig. 7B, C, D, E). JAZF1, formally recognized as Juxtaposed with Another Zinc Finger Gene 1, known by the aliases Tip27 and ZNF802, emerges as a versatile regulatory factor initially identified as a corepressor of the orphan nuclear receptor TR4 (NR2C2). JAZF1 co-localizes with TR4 in the nucleus and engages explicitly with the ligand-binding domain of TR4 [33, 34]. TR4 has been shown to influence metabolic processes, such as gluconeogenesis, adipogenesis, and inflammatory responses, and promote VEGF expression [35–37]. Our investigation substantiates that the overexpression of JAZF1 exerts inhibitory effects on the proliferation, migration, vascular formation, and VEGFA expression in HUVECs under conditions of elevated glucose.

## Methods

### Extraction and Identification of ADSC-Exos

Adipose tissue was taken from the discarded adipose tissue of surgical patients. Informed consent was obtained from the patient and approval was obtained from the ethics committee. ADSCs were extracted and cultured following previously described methods [38]. ADSCs were cultured with lipogenic, osteogenic, and chondrogenic differentiation media (OriCell) to assess their differentiation potential. After 21 days, oil red O, alizarin red, and algal blue staining were performed, and images were captured using a light microscope (Olympus). ADSC-Exos were isolated as follows: Centrifuge the cell culture supernatant at 300 g for 15 min to remove cellular debris effectively. Retrieve the resulting supernatant and subject it to a subsequent centrifugation at 2000 g for 15 min. Subsequently, subject the resulting supernatant to a centrifugation at 10000 g for 30 min. Conduct a centrifugation at 100000 g for 70 min on the obtained supernatant, followed by discarding the resulting supernatant. Resuspend the pellet in phosphate-buffered saline (PBS, Gibco) and repeat the centrifugation at 100000 g for an additional 70 min. The resulting pellet represents the isolated exosome. Exosome size distribution was determined using nanoparticle tracking analysis (NTA, ZetaView), and their morphology was observed by transmission electron microscopy (TEM, Thermoscientific Talos). Western blotting was conducted to detect exosome markers, including CD9, HSP70, TSG101 (Affinity) and calnexin (Abcam).

### Exosome Uptake

To assess the uptake of ADSC-Exos by endothelial cells, we utilized HUVECs as the cellular model. HUVECs were procured from OirCell. The cells were cultured in 89% DMEM with a glucose concentration of 4.5 g/l (Gibco), supplemented with 10% exosome-free serum (Epizyme Biotech), and 1% penicillin–streptomycin (Beyotime). ADSC-Exos, pre-labelled with the red fluorescent dye PKH26 (Sigma), were co-cultured with HUVECs for 24 h. After two PBS washes, cells were fixed with 4% paraformaldehyde for 20 min, then stained with FITC Phalloidin (Solarbio) for 1 h. Hoechst 33,342 (RIBOBIO) stains cell nuclei for 20 min. The fluorescence was visualized using a laser-scanning confocal microscope (Nikon A1).

### CCK‑8 Assay

HUVECs (5 × 10^3^ cells per well) with different treatments (transfected or not) were seeded in 96-well plates and cultured overnight. The culture medium was replaced with a conditioned medium. After 24 or 48 h, CCK-8 (Dojindo) reagent was added, and the absorbance at 450 nm was measured using a SpectraMax M5e.

### EdU Incorporation Assay

HUVECs (5 × 10^3^ cells per well) with different treatments were seeded into 96-well plates and co-cultured with ADSCs-Exos for 24 h. The culture medium of HUVECs was replaced with EdU medium (RIBOBIO) and incubated for 2 h. The cells were washed with PBS, fixed with 4% paraformaldehyde, and permeabilized with 0.5% Triton X-100. Apollo staining was performed for 30 min, followed by Hoechst 33,342 nucleus staining. Finally, the staining was observed and photographed under a fluorescence microscope (Olympus).

### Wound Healing Assay

HUVECs were incubated with different treatments in 6-well plates. Wounds were created using a 200μL micropipette tip. The cells were treated with conditioned media. Images of each scratch were captured at 0 h and 24 h using an optical microscope (Olympus). Wound Closure Rate = (Original Wound Area − Unhealed Wound Area)/Original Wound Area × 100%.

### Transwell Assay

HUVECs with different treatments were suspended in a medium without FBS. 3 × 10^3^ cells were seeded in the upper chamber of a 24-well transwell system with 8.0 μm pore-sized filters (Corning), and 600μL of the conditioned medium was added to the lower chamber. After 24 h of incubation, the migrated cells on the lower surface of the filter were fixed with 4% paraformaldehyde for 30 min and then stained with crystal violet (Beyotime). The migrated cells were observed and quantified under an optical microscope (Olympus).

### Tube Formation Assay

24-well plates were pre-coated with Matrigel (Corning). 5 × 10^4^ HUVECs per well with different treatments were seeded in culture plates. After 6 h of culture, tube formation was observed under the microscope (Olympus). The Total tube length was calculated using Image J software.

### High-Throughput Sequencing of miRNAs

When the HUVECs reached a confluence of 60% to 70%, they were subjected to co-culture with 200 μg/ml ADSC-Exos for 24 h. After total RNA extraction using the Trizol reagent (Invitrogen), RNA molecules in the size range of 18–30 nt were enriched through polyacrylamide gel electrophoresis. Subsequently, the 3’ adapters were added, and the 36–44 nt RNAs were further enriched. The 5’ adapters were ligated to the RNAs as well. The ligation products underwent reverse transcription by PCR amplification, and the 140–160 bp PCR products were enriched to generate a cDNA library that was subsequently sequenced using Illumina Novaseq6000 (Gene Denovo Biotechnology Co). For miRNA identification, mirbase (Release 22) was employed. Differentially expressed miRNAs were filtered based on fold change (FC) and *P*-value criteria (*p* < 0.05 and FC > 1.5). TargetScan (http://www.targetscan.org/), StartBase (http://www.startbase.com/) and miRTarBase (https://mirtarbase.cuhk.edu.cn/) were utilized to investigate the target genes of miRNAs and the conserved sites bound by the seed region of miRNAs.

### Quantitative Real-Time PCR (qRT-PCR) Analysis

To detect miRNA and mRNA expression, reverse transcription was conducted using the Mir-X™ miRNA First-Strand Synthesis Kit and the PrimeScript™ RT Master Mix (Takara), respectively. The quantification of miRNA and mRNA was performed using the TB Green® Premix Ex Taq™ II (Takara) in qRT-PCR assays. U6 and GAPDH were employed as internal miRNA and mRNA quantification controls, respectively. The primer sequences used in the qRT-PCR are provided in Table S1.

### Cell Transfection

The miR-146a-5p mimics, mimics NC, miRNA-146a -5p inhibitor, inhibitor NC, vector, and pcDNA3.1 JAZF1 were procured from GenePharma Biotechnology. Following the manufacturer’s instructions, the transfection was performed using Polyplus reagent (Polyplus Transfection® SA).

### Luciferase Activity Assays

Mutant versions of the miR-146a-5p binding site sequences in JAZF1 were synthesized and inserted into the psiCHECK2 luciferase reporter vector (Hanbio Biotechnology) to create JAZF1 wild-type (JAZF1-WT) and mutant (JAZF1-Mut). The dual-luciferase assay was conducted using a dual-luciferase assay kit (Hanbio Biotechnology). The relative luciferase activity of fireflies and kidney worms was measured using SpectraMax M5e.

### Western Blotting

Cells were lysed in RIPA lysis buffer containing protease inhibitors, followed by incubation on ice for 30 min. After centrifugation at 12,000 g for 30 min at 4 °C, the supernatant was mixed with loading buffer and heated at 100 °C. The proteins were separated by electrophoresis and transferred onto a polyvinylidene difluoride membrane (Millipore). Subsequently, the membranes were blocked with skim milk for 1 h. They were then incubated overnight at 4 °C with primary antibodies against β-actin (Affinity), VEGFA (Abcam), and JAZF1 (Santa Cruz Biotechnology). Afterwards, the membranes were incubated with secondary antibodies (Biosharp) at room temperature for 1 h for immunoblotting. Finally, the proteins were visualized using ECL chemiluminescence reagents (Thermo Fisher Scientific).

### Characterization of HAMA

HAMA (EngineeringForLife) was dissolved at 37 °C, and 500 μg of ADSC-Exos was added. UV chemical cross-linking was performed for 5 s to create the composite hydrogel. The composite hydrogel was immersed in PBS in a 24-well plate, and surface supernatants were collected at specific time points (2d, 4d, 6d, 8d, 10d, 12d, 14d, 16d, 18d, 20d) and transferred to fresh PBS. The number of released exosomes was measured using the BCA Protein Analysis Kit (Beyotime), expressed as a percentage of release. To assess hydrogel degradation, weights were measured after removing surface supernatant at specified time points (2d, 4d, 6d, 8d, 10d, 12d, 14d, 16d, 18d, 20d). Degradation rate = weight of the sample at a specific time point/initial weight of the sample × 100%.

### Animal Models

Male C57/BL mice (8 weeks old, 20 ± 2 g) were obtained from Anhui Medical University. Diabetes mellitus was induced in healthy mice through a high-sugar and high-fat diet and intraperitoneal injection of streptozotocin (STZ, 40 mg/kg, Sigma). The mice, subjected to weekly injections of STZ over a span of four to five consecutive weeks, manifested random blood glucose levels surpassing 16.7 for three successive days, conclusively affirming the efficacy of the modeling procedure. A full-thickness skin wound measuring 10 mm was excised at the midline of the mouse’s back. An intraperitoneal injection of 1.25% Avertin sodium (0.2 ml/10 g, Easycheck) was administered for anesthesia. To investigate the metabolism and distribution of ADSC-Exos, six C57/BL mice were randomly selected and divided into two groups. One group was subcutaneously injected with PKH26-labeled ADSC-Exos (PKH-Exos), while the other group was treated with HAMA combined with PKH26-labeled ADSC-Exos (HAMA-PKH-Exos). The exosome changes were observed using the Animal In Vivo Imaging Instrument (AniView100) at postoperative time points: 0d, 1d, 3d, 5d, and 7d. The remaining mice were randomly assigned to three groups (*N* = 5 per group): DW, Exos, and HAMA-Exos. Digital photographs of the wounds were captured on 0d, 3d, 7d, 10d, and 14d.

### Hematoxylin and Eosin (H&E), Masson’s, Immunohistochemical (IHC), and Immunofluorescence (IF) Staining

After 14 days, skin samples from the wound area were collected and fixed with 4% paraformaldehyde for 48 h. Dehydration was carried out, and samples were embedded in paraffin and cut into 5 μm serial slices. Histological features were assessed using H&E and Masson’s trichrome staining (Sigma). For IHC and IF, sections were incubated with primary antibodies against collagen I (Immunoway) and VEGFA (Abcam) at 4 °C overnight. Subsequently, incubation with secondary antibodies (Servicebio) was performed for 1 h at room temperature. Images were captured using fluorescence microscopy (ZEISS).

### Statistical Analysis

In this study, data analysis was performed using GraphPad Prism 8.0.1 software. The images were analyzed using Image J. Comparisons of two or more groups were performed using Student’s t-test or one-way ANOVA. The results are expressed as the mean ± standard deviation (SD), and a significance level of *P* < 0.05 was considered statistically significant.

### Supplementary Information

Below is the link to the electronic supplementary material.

Supplementary file3 (DOCX 1558 KB)

## Data Availability

The data from this study are available from the corresponding author upon reasonable request.
