# Supplementary material for: Exosomes Derived from Adipose Stem Cells Enhance Angiogenesis in Diabetic Wound Via miR-146a-5p/JAZF1 Axis
Source: Stem Cell Rev Rep. 2024 Feb 23;20(4):1026–39. doi: 10.1007/s12015-024-10685-8 (PMC11087353; doi:10.1007/s12015-024-10685-8)
Supplement: Supplementary file 1 — Supplementary file3 (DOCX 1558 KB) [file 12015_2024_10685_MOESM1_ESM.docx]

**Supplementary information**


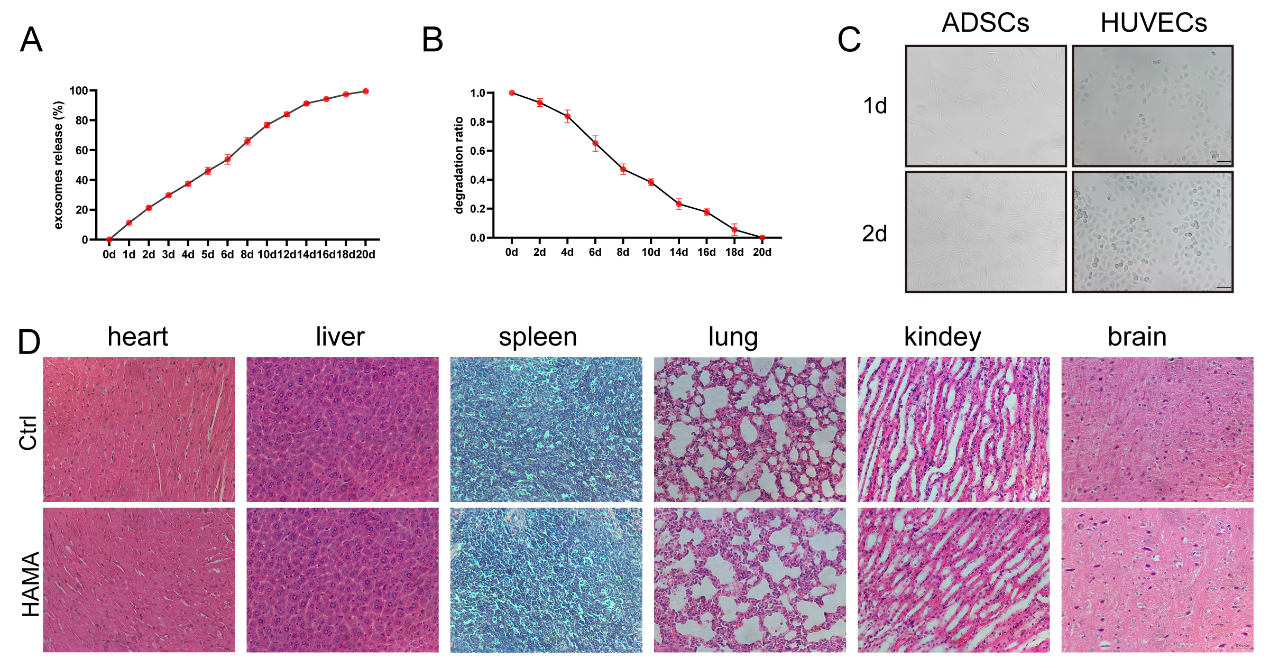


**Figure S1.** Biological properties and safety of HAMA. **A** Exosome release. N=4. **B** HAMA degradation. N=4. **C** HAMA in vitro 3D culture of ADSCs and HUVECs. N=3. Scale bar:50μm. **D** HE staining of each group's heart, liver, spleen, lung, kidney, and brain tissue sections for safety evaluation. N=3. Scale bar:50μm. Data are presented as mean ± SD.


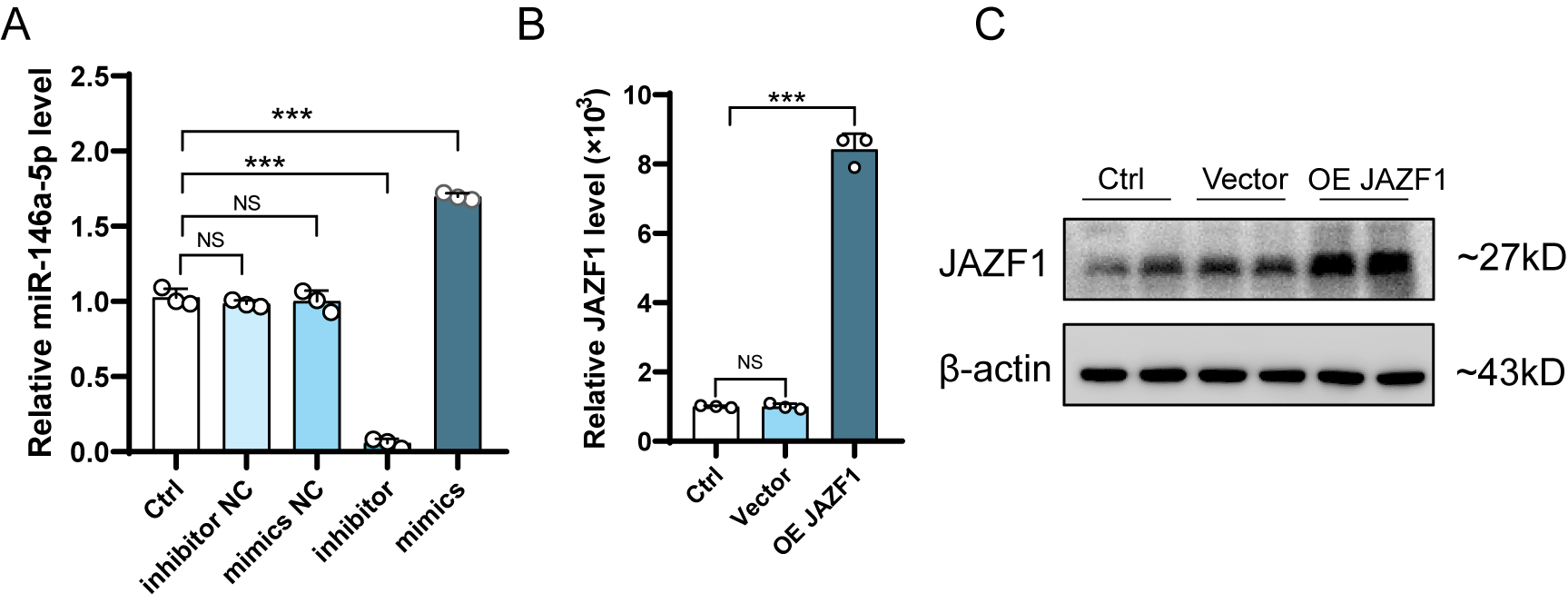


**Figure S2.** Transfection efficiency of miR-146a-5p and JAZF1. **A** Expression of miR-146a-5p after transfection with miR-146a-5p mimics, mimics NC, miR-146a-5p inhibitor, and inhibitor NC in HUVECs, respectively. N=3. **B** Expression of JAZF1 mRNA after transfection with vector and JAZF1 pcDNA3.1 in HUVECs. N=3. **C** Expression of JAZF1 protein after transfection with vector and JAZF1 in HUVECs. N=4. Data are presented as mean ± SD. *p < 0.05, **p < 0.01, ***p < 0.001.

Table S1. miRNA and mRNA sequences

| RNA | 5’→3’ |
| --- | --- |
| miR-143-3p | TGAGATGAAGCACTGTAGCTC |
| miR-146a-5p | TGAGAACTGAATTCCATGGGTT |
| miR-155-3p | CTCCTACATATTAGCATTAACA |
| miR-796-3p | CTGGGATCTCCGGGGTCTTGGTT |
| miR-939-5p | TGGGGAGCTGAGGCTCTGGGGGTG |
| IRAK1 | F: GCACCCACAACTTCTCGGAG  R: CACCGTGTTCCTCATCACCG |
| HNRNPD | F: GCGTGGGTTCTGCTTTATTACC  R: TTGCTGATATTGTTCCTTCGACA |
| KCTD15 | F: TCCTCGCTTCACACACACG  R: GCACAGGTGCATTGGACTTG |
| TMEM136 | F: TTCTTAGGGTGCATCCTTTGTTT  R: TCATAGCTTCGGTGCTTATTCAG |
| JAZF1 | F: AGGCAGCGAGTATGACGAG  R: TCGGAGCTGATGGCACTCT |
| GAPDH | F: CTGGGCTACACTGAGCACC  R: AAGTGGTCGTTGAGGGCAATG |
